# A Framework for Enhancing Access to Equitable Home Care for 2SLGBTQ+ Communities

**DOI:** 10.3390/ijerph17207533

**Published:** 2020-10-16

**Authors:** Andrea Daley, Shari Brotman, Judith A. MacDonnell, Melissa St. Pierre

**Affiliations:** 1 School of Social Work, Renison University College (at University of Waterloo), Waterloo, ON N2L 3G4, Canada; 2 School of Social Work, McGill University Montreal, Montreal, QC H3A 2A7, Canada; shari.brotman@mcgill.ca; 3 School of Nursing, York University, 422 Health, Nursing & Environment Studies, Bldg, 4700 Keele Street, Toronto, ON M3J 1P3, Canada; jmacdonn@yorku.ca; 4 Knowledge Mobilization, Supply Chain Advancement Network in Health, Odette School of Business, University of Windsor, Windsor, ON N9B 3P4, Canada; Melissa.Stpierre@uwindsor.ca

**Keywords:** lesbian, gay, bisexual, transgender health, queer and trans health, two-spirit health, health services access and equity, health inequities, equity, diversity, and inclusion, gender-based analysis, health policy, organizational change

## Abstract

Canadian, US, and UK public health and clinical research has identified barriers to health service access for Two-Spirit, lesbian, gay, bisexual, transgender, queer, non-binary, and intersex (2SLGBTQ+) communities. While offering important insight into the health service experiences of 2SLGBTQ+ communities, this body of research only recently, and still only minimally, reports on home care access experiences. Drawing on key findings from the 2SLGBTQ+ Home Care Access Project, a mixed-methods, Ontario-wide study, this paper animates an Access and Equity Framework, using participant stories and perspectives to underscore the relevance and effectiveness of the Framework as a tool to support systematic organizational assessment, evaluation, and implementation of access and equity strategies. Home care organizations can use this tool to assess their programs and services along a continuum of intentionally inviting, unintentionally inviting, unintentionally disinviting, and intentionally disinviting care for 2SLGBTQ+ people. To support this process, the framework includes six indicators of access to care: community engagement, leadership, environment, policies and processes, education and training, and programs and services.

## 1. Introduction

Equity and accessibility as important principles of social policy and institutional practice have gained the attention of the federal government in Canada, which has begun to organize and promote criteria to evaluate initiatives funded by them (including, most notably, in the areas of federal employment, research, and post-secondary education) through the lens of equity, diversity, and inclusion (EDI). There is currently a movement across Canada to operationalize EDI and to provide a framework for initiating policy analysis and evaluation [1]. While health and social care policy has not historically been a primary focus of EDI initiatives at the federal level, in part because health and social care policy is a provincial responsibility, it has become increasingly evident over the past several months that the need for attention to access and equity in the design and delivery of health and social care services, and for federal involvement in policy planning, is urgent. The recent COVID-19 pandemic has exposed significant gaps in Canada’s healthcare system with respect to issues of access and equity barriers for marginalized communities, including Two-Spirit, lesbian, gay, bisexual, queer, transgender, non-binary, and intersex (2SLGBTQ+) communities, and particularly those whose intersectional identities across various social locations (race, class, gender identity, ability, age, etc.) have resulted in lifelong exposure to discrimination resulting in significant health disparities [2,3].

One important policy development in Canada is the introduction of gender-based analysis plus (GBA+) as a means of operationalizing principles of EDI through an intersectional lens [4]. Status of Women Canada was first tasked with managing GBA by the federal government, who officially committed to adopting it as early as 1995. In 2015, the Status of Women Canada introduced GBA+ to enhance the capacity for federal organizations (and other organizations interested in developing EDI policies) to move beyond only accounting for gender equity in government policy, legislation, and regulations. Rather than simply attending to binary gender categories (as was the focus of the previous GBA policy model introduced in 2005 by the federal government of Canada), the Status of Women presented the GBA+ model, which centres intersectionality as a lens through which to address gender equity in conjunction with other categories of difference, including, but not limited to gender identity and sexual orientation [5,6].

Despite the emergence of GBA and GBA+ in Canadian policy analysis, by 2016, only 29 of approximately 110 federal organizations had committed to it as a process for policy/program evaluation and implementation [7]. According to Hulko et al. [4], an important critique of EDI and GBA+ relates to the challenges in operationalizing the model across multiple categories of difference, and attending to corresponding forms of interlocking oppression simultaneously, with current assessments of the model suggesting that some identity categories may become prioritized over others for several reasons. This has raised concerns among feminist, antiracist, and 2SLGBTQ+ activists and advocates that the realities of all marginalized people, including diverse racialized and gender and sexual minority groups may become minimized or ignored in the policy agendas of some institutions, particularly when there is no attention to issues of representation of these groups at the decision-making table [7,8,9,10,11]. In addition, institutions in which there is no, or little incentivizing for change, limited resources directed toward both implementation and evaluation, and an absence of support from management may be unmotivated to undertake access and equity evaluations and to adapt practices [7,10]. Importantly, scholars have noted that decades of entrenched austerity and governmentality limit the actualization of GBA+ across sectors of government. Despite these limitations, the momentum for engaging in policy and practice level change to enhance equity, diversity, and inclusion is, nonetheless, promising. 

Although federal and provincial governments have applied a variety of forms of gender-based analysis to evaluate equity initiatives in diverse sectors of government oversight, cross-jurisdictional applications have lacked a unified vision or strategy [12], particularly with respect to 2SLGBTQ+ people [9]. And while the federal government’s Standing Committee on Health launched a nation-wide consultation in 2019 to gather information on how to best include LGBT+ populations in education, research, policy and practice, the findings do not fully attend to intersectional realities within and across queer and trans communities. In addition, critical analysis of the report cautions against the hetero-cis normative ideology of requiring 2SLGBTQ+ people to “fit into” pre-existing structures embedded within it [13]. In light of these critiques, the usefulness of the GBA+ framework to advance change for 2SLGBTQ+ communities in health across Canada remains uncertain and largely undocumented [9]. Finally, a significant critique of EDI/GBA+ at the current time is the top–down approach utilized in the change-making process, with administrators and “gender experts” called upon to develop strategic action plans without requiring inclusion of activists and people with lived experience at the decision-making table where policy mandates are operationalized [11,14]. This has a detrimental impact on the potential for change to redress homophobia, transphobia, and hetero-cissexism [9].

### 1.1. 2SLGBTQ+ Health Services Access and Equity

Scholarship addressing the significant access and equity barriers to health and social care services faced by 2SLGBTQ+ populations across the globe has proliferated over the past twenty years. We now have a much deeper understanding of some of the issues of 2SLGBTQ+ access and equity within health and social care services in Global North societies, with the growing body of research on access to care pointing to the detrimental impact of inequities on the health and well-being of 2SLGBTQ+ individuals and communities. A diversity of topics has been addressed within this scholarship and includes attention to challenges experienced across the life course such as those related to aging [15,16,17]; gender identity [18,19,20]; racialization [21,22,23]; Two-Spirit identity [24,25] health conditions [26,27]; rurality [28,29,30]; and health services policy and delivery [31,32].

Collectively, this vast body of research indicates deleterious health disparities among, and harms to, 2SLGBTQ+ individuals associated with both marked visibility and invisibility during care interactions as a result of institutionalized heterosexism, homophobia, biphobia, and cissexism and transphobia. While offering important insight into the health service experiences of 2SLGBTQ+ communities, this body of research on access and equity barriers in health and social care services within these communities only recently, and still only minimally, reports on home care experiences [33,34,35,36,37,38]. For example, Grigorovich [35,36] explores quality of care related to service provider knowledge of, and comfort with, sexual diversity and the decision-making processes related to disclosure of older lesbian and bisexual women during interactions with home care service providers, citing experiences of overt and subtle heterosexism and discrimination. References to home care in 2SLGBTQ+ health-related literature have often been anecdotal and/or represent the perspectives and insights of service providers rather than sexual and gender minority people themselves [31,38,39,40,41,42].

Conversely, home care research on practice issues related to access and equity (e.g., cultural competency) has not included 2SLGBTQ+ populations as a social group [40]. Consequently, we know very little about how 2SLGBTQ+ communities experience home care services or their perspectives on the degree to which home care service providers are equipped to understand their unique concerns related to health needs and care interactions. Brotman et al. [43] describe how receiving care in the home is unique for 2SLGBTQ+ people, highlighting that older adults who experienced particularly acute forms of violence and oppression in their younger adult lives often experience home as one of the few spaces in which they can live out their identities and relationships in safety and security. Given what is known about 2SLGBTQ+ communities and barriers to accessing equitable health services, and the significance of “home” for members of these communities, the lack of existing research on home care is an important social justice concern in need of further attention [28,44].

### 1.2. Organizational Responses to 2SLGBTQ+ Health Services Access and Equity

Policy and program advocacy in Canada emerging in parallel with this body of 2SLGBTQ+ health services research has been driven by strong and vocal health advocacy movements within diverse 2SLGBTQ+ communities and is supported by allies within the health and social care service sectors across Canada. Nonetheless, despite decades of effort and many signs of improvement in several jurisdictions and localized sites, uptake for change has largely been fragmented across the health and social care service system. Underlying organizational assumptions and ideological commitments to access and equity of health services do not often include sexual and gender minority communities. Often, these communities may be only minimally and unevenly included in access and equity initiatives [45].

In some jurisdictions, most notably in the large urban centres of Toronto and Vancouver, change has led to the development of 2SLGBTQ+-specific healthcare organizations funded by provincial governments (for example, LGBTQ primary healthcare team, Sherbourne Health Centre, Toronto; Rainbow Services, Centre for Addiction and Mental Health, Toronto; Transgender Health Program, Vancouver Coastal Health, Vancouver), while in other locations, such as in Quebec, provincial ministries of health and social services have funded provincial initiatives to improve access to education and training among frontline service providers (for example, Rainbow Health Ontario, Toronto; Prism, Vancouver; Quebec Ministry of Health and Social Services, in partnership with the Seniors Secretariat and the CIUSSS-CHUS Estrie online platform (April 2019–March 2021)). In many communities across the country, including small city and rural settings, service or training programs have been spearheaded by local 2SLGBTQ+ advocacy and service delivery organizations, often in partnership with mainstream health service deliverers, and have succeeded in improving access and equity in specific health and social care organizations. Still, there continues to be a lack of recognition of the unique realities and needs of 2SLGBTQ+ citizens on the part of health and social care service organizations, decision-makers, and providers. The denial of the historic and current barriers faced by both individuals and communities in accessing appropriate and relevant care remains a significant problem. It is important to emphasize that the diversity of community realities and needs, as well as the difference in specific organizational and institutional mandates across sector services, renders it challenging to develop intersectoral policies and requires attention to change processes that are sensitive to specificity. An understanding of the way in which access and equity is experienced by diverse 2SLGBTQ+ communities, however, remains a necessary precursor to advancing a change agenda that is equitable, transferable, universally applied, and evaluated across jurisdictions and sectors.

To this end, this paper highlights the *Home Care Access & Equity Framework* (herein referred to as the A&E Framework) developed as part of a research project on the experiences of home care access among 2SLGBTQ+ populations in the province of Ontario [46]. We describe the A&E Framework in detail, providing insights into its development and process for implementation as a tool, which may be useful to enacting change within home care settings specifically and in health and social care service organizations generally. Drawing on survey data results and participant narratives, this paper animates the A&E Framework, underscoring its relevance and effectiveness as a tool to support home care organizations to evaluate their existing policies and practices in relation to service access and equity for 2SLGBTQ+ populations and to identify and implement strategies for change to improve programs and policies. While the paper centres on identity categories of sexual orientation and gender identity, we facilitate an intersectional consideration of health services access and equity for 2SLGBTQ+ communities. Similarly, notwithstanding the focus of the A&E Framework on home care access and equity, we examine its transferability and utility to other health care service contexts.

### 1.3. The 2SLGBTQ+ Home Care Access Project

In response to what is known about 2SLGBTQ+ communities and barriers to accessing health services, and the significance of the home for members of these communities, we undertook a provincially-based project that explored home care access experiences for 2SLGBTQ+ people in Ontario, Canada. The overarching goal of our project was to expand the breadth and depth of knowledge about 2SLGBTQ+ home care access to inform sectoral policy and practice change to enhance equitable access to high quality home care. A central analytic goal and knowledge mobilization strategy of this project was the development of the A&E Framework designed to operationalize findings into a concrete tool of policy and program evaluation and change, including the identification of key areas for consideration in the redesign of policies and services, as well as the elaboration of clear processes for engaging in change at all levels of organizational practice. Please note that at the time of the project, publicly funded home care in Ontario was coordinated by 14 Community Care Access Centres (CCACs) representing different geographic locations across Ontario [38]. However, June 2017, Ontario transferred the responsibility of home care service delivery to its 14 Local Health Integration Networks (LHIN). While this project was contextualized and implemented within the former structure of home care delivery in Ontario, the delivery of home care services remains the same. That is, direct care continues to be delivered by a range of health care providers employed by home care agencies that are contracted by a centralized body (LHIN). As such, within the context of a differently structured home care delivery, we suggest that the project findings presented in this report are relevant as they reflect the experiences of 2SLGBTQ+ service users and home care service providers.

## 2. Methodology

Our research was guided by the transformative paradigm as an ethical lens that problematizes the social and political context of inequitable health services distribution and the impacts of oppressive institutional practices on 2SLGBTQ+ communities [47]. As a social justice seeking endeavour, our research objectives, design, and process emulated the ethical assumption of the transformative paradigm “that emphasizes the pursuit of social justice and the furtherance of human rights” ([48] p. 22) for home care seeking and receiving 2SLGBTQ+ people, relying upon “useful knowledge” produced through both research process and outcomes [47,49,50]. Of particular importance was the development of a research design and methods that centre the voices of marginalized communities to address research gaps in home care [47]. This included ensuring that decision-making processes and procedures fundamentally included 2SLGBTQ+ people through the development of Community Advisory Committees. Our Community Advisory Committees (CAC) included 2SLGBTQ+ community members with a range of relationships with the home care sector (service users, service providers, administrators) and diverse experiences of disability, race, Two-Spirit identity, and age, as well as representatives from 2SLGBTQ+-specific services, disability and seniors services, and home care provider organizations (service providers and administrators). A second CAC served as a specific “older adults” lens. 2SLGBTQ+ seniors and 2SLGBTQ+ community members affiliated with senior health and social services participated. The CACs were involved in developing and implementing data collection tools (i.e., surveys), a service user recruitment strategy, as well as a knowledge mobilization plan to engage 2SLGBTQ+ communities, home care service providers and organizations, and policy decision-makers. The research team also explored preliminary findings with each CAC, seeking guidance in the direction of further analysis.

We sought to: (1) provide quantitative and qualitative evidence of 2SLGBTQ+ home care service use and home care administrators’ and providers’ organizational knowledge about service provision to 2SLGBTQ+ people; (2) foster dialogue and elicit multiple narratives on 2SLGBTQ+ service access from individuals who are differently situated to home care; and (3) create and sustain collaborative relationships with community “knowers” for the purpose of knowledge co-production. To this end, we adopted a community-engaged, participatory, mixed-methods approach [51], using a combination of surveys, individual interviews, and focus groups to facilitate community conversations with identified stakeholder groups over a four-year period.

The project was operationalized across two phases. Phase 1 consisted of: (a) a synthesis and critical discourse analysis of the “key indicators” literature on access and equity in health care organizations informed by a gender-based diversity analysis [45]; and (b) key informant interviews with 2SLGBTQ+-positive health service organizations that explored experiences, opinions, and insights into the history and development of established agencies providing service to members of 2SLGBTQ+ communities [52]. Phase 2 included: (a) 115 surveys and 38 semistructured interviews with 2SLGBTQ+ people using or having a history of using home care services; (b) 379 surveys and 19 individual and focus group interviews with home care service providers; (c) 6 interviews with senior administrators in regional home care access centres; and (d) 12 interviews with key informants (i.e., home care referral sources). The four cohorts were identified in order to get a wide-angle understanding of key issues and challenges from all constituent groups who have a stake in the design and delivery of home care to 2SLGBTQ+ communities and who represent distinct and diverse perspectives on access and equity. However, for the purpose of this paper, we draw on key findings from the 2SLGBTQ+ service user, home care service provider, and home care administrator surveys, interviews, and focus groups. Findings are reported here insofar as they support the development and usefulness of the A&E Framework, demonstrate and justify the need for such a Framework to advance change agendas, and provide insights into the Framework and its components. The study was conducted in accordance with the Declaration of Helsinki; ethics clearance was sought through and received by all participating institutions before the study began (e2011-128).

This section briefly outlines the methodology of the project for the purposes of providing necessary information about the study parameters and processes with a specific focus on 2SLGBTQ+ service user and service provider participants. For a complete discussion of the full recruitment strategy, data collection, and participant demographics for each cohort, please refer to the project report, LGBTTQI Communities and Home Care in Ontario: Project Report.

### 2.1. Recruitment and Data Collection

#### Surveys, Interviews, and Focus Groups

Distinct recruitment strategies were developed for each participant cohort, focussing on a combination of strategies including snowball sampling, outreach via the media and local organizations, professional networks, and community partners. The procedures for survey completion were identical for the service user and service provider participants. Two anonymous web-based surveys—Service User Survey (SUS) and Service Provider Survey (SPS)—were designed using the software program Fluid Surveys. Participants had the option of completing a paper version of the surveys, and both were available in English and French. Informed consent was received by each participant prior to the completion of the survey. At the end of each survey, participants were asked whether they would be interested in participating in an individual interview or focus group to discuss further their experiences as a user or provider of home care. (Details about the survey items and interview schedules can be found in the LGBTTQI Communities and Home Care in Ontario: Project Report). Electronic data were stored in password-protected files on a secure Canadian server, and participants’ contact information was stored separately from their survey responses to ensure anonymity.

Participants were offered the option of completing the interview/focus group in English or French. The service user, service provider, and home care administrator interviews/focus groups ranged in length from 45 min to 1.5 h. Participants provided consent for participating in the interview/focus group and audio-recording the conversation before the start of each interview/focus group.

### 2.2. Data Analysis

#### 2.2.1. Quantitative Data

Quantitative analyses were facilitated through computer software, namely SPSS. Descriptive statistics (e.g., frequency counts, percentages, and measures of central tendency) were run to describe participant groups, in addition to key aspects of their experiences. Bivariate analyses (e.g., correlations, *t*-tests, Chi-Square tests) were also conducted to explore possible associations between variables.

#### 2.2.2. Qualitative Data

The audio files of all individual interviews and focus groups were transcribed verbatim by a professional transcriptionist and subsequently anonymized in preparation for analysis. Analysis was undertaken in an iterative process, moving between data collection and analysis. We used a critical lens reflecting our transformational analysis objective to identify themes emerging from the qualitative data. We incorporated theoretical concepts embedded in the intersectional life course perspective [53] and categories outlined within the GBA+ approach to enhance our analytic capacity to pay attention to how the lived experience of events (particularly those related to access and equity) are structured through institutional policies and practices at the local level. A minimum of two researchers independently coded each transcript line by line (first level codes). Using focused coding and the constant comparative method from grounded theory, first level codes were further refined and organized into categories [54]. Theoretical coding was implemented as a means of linking the codes that emerged from the process of focused coding, and further developing the relationship between categories. In keeping with a grounded theory approach, memo-writing was performed in an effort to elaborate processes, assumptions, and actions throughout the data analysis process. Coding was facilitated through the use of Nvivo qualitative analysis software. Steps were taken to ensure that neither individuals nor organizations are identified in the reporting of findings. In a final step in our analysis, themes were “structurally” coded, through a process of connecting perspectives and stories to institutional practices and policies, in order to illuminate how policy and practices either limit or enhance access and equity, and to identify components necessary to account for in a framework for change.

### 2.3. Participants

Overall, over half of the 2SLGBTQ+ home care service user participants were under the age of 50; nearly 50% were single and living with a range of disabilities. Fifty percent of the service users reported incomes at or below the poverty line, and 1 in 10 had precarious housing. Participants identified as identified as gay (37%), lesbian (30%), queer (18%), bisexual (15%), and Two-Spirit (10%). Forty-six percent identified as female, with other participants identifying as male (39%), as Two-Spirit (10%), and 20% were trans and/or had a history of transitioning sex/gender. Twenty-seven percent were perceived or treated as a racialized person or person of colour. The characteristics of the interview participants were almost identical to the sample of service users described above, with most self-identifying as White, male or female, and gay or lesbian.

The majority of home care service provider participants were nurses (33%), personal support workers (23%), and care coordinators from home care access centres (24%). Over 50% had worked for five years or less in home care. Ten percent of service provider survey participants identified as 2SLGBTQ+ (see the LGBTTQI Communities and Home Care in Ontario: Project Report for more demographic details).

Our interviews with senior administrators from six regional home care access centres explored home care access and equity from an organizational perspective. The six access centres, with a mandate to coordinate home care access across a region, were geographically diverse in that they served urban, rural, and suburban regions of the province. To protect their identities given their specific leadership roles, we did not collect demographic information on the administrators (Please note that the content of this paragraph is reprinted with permission of the authors from our project report [46].

#### 2.3.1. 2SLGBTQ+ Home Care Access and Equity Framework

**Figure 1:**
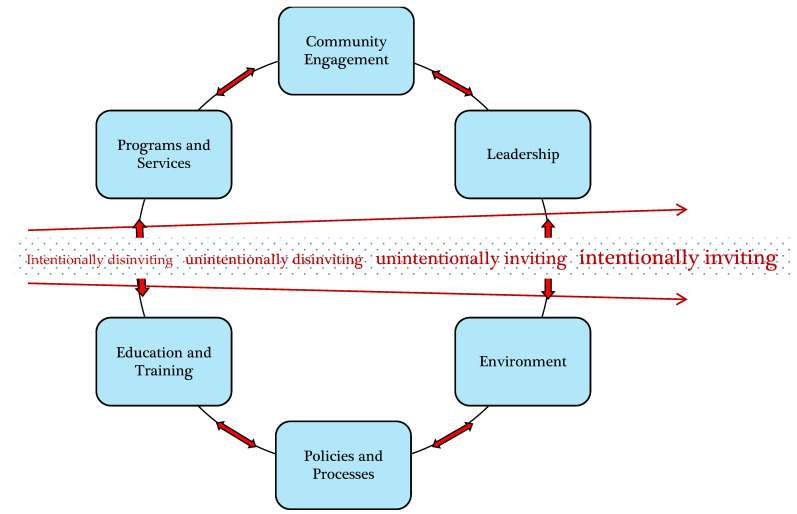
Two-Spirit, lesbian, gay, bisexual, transgender, queer, non-binary, and intersex (2SLGBTQ+) home care access and equity framework.

The Home Care Access and Equity Framework (see Figure 1) takes into account the stories and perspectives of our participants through the articulation of the unique dimensions of care described in the data related to the provision of services in the home. Attention to the nature and complexity of coordinating care in the home, the reality that there is a range of regulated and unregulated health care providers who work for multiple contracted not-for-profit and for-profit service provider agencies in the home, and the reality of austerity measures which serve to limit options for care were all factors which influenced the experiences of home care among participants and served to shape the development of the A&E Framework. Themes emerging from the accounts of participants including those related to personal (identities and histories), relational (interactions and relationships between service users and service providers), and structural (regulatory, eligibility, policy, and environmental) features addressed by participants formed the basis of the A&E Framework.

The A&E Framework integrates two central components. The first component is an invitational continuum adapted from invitational theory [55,56], and which includes four points: intentionally disinviting, unintentionally disinviting, unintentionally inviting, and intentionally inviting. The *intentionality* of the dynamic at both the individual service provider and organizational levels is key to understanding how to move toward consistently respectful and inviting or affirming care. For organizations to work toward creating *consistently inviting or affirming care* to meet the needs of diverse service users, providing training is an important first step. Individual service providers need to understand key elements of affirming practice in order to consistently provide respectful and affirming care. However, if service users anticipate or experience exclusion at the organizational level with unwelcoming environments, discriminatory policies, and/or exclusionary intake forms, this disconnect between the individual service provider and organization dynamics contributes to unintentionally disinviting or non-affirming care. Service providers or organizations may at times provide affirming care; however, unless they understand the complexity of dynamics which shape consistently affirming care, care is unintentionally inviting and the organization lacks awareness of how to improve the quality of care [46].

The second component is six indicators of access to care for 2SLGBTQ+ people: community engagement, leadership, environment, policies and processes, education and training, and programs and services. Each of the six indicators has evaluation prompts that take into account dynamics of power and privilege that can assist organizations to undertake systematic self-assessments of their policies, programs, and services along the invitational continuum and to consider change strategies to enhance their capacity to provide consistently inclusive, affirming care for 2SLGBTQ+ people—that is, intentionally inviting care. As integrated components, the invitational continuum and the six indicators of access to care are implemented vis-à-vis the A&E Framework to reflect how service users might perceive and/or anticipate care as inviting or disinviting, as well as factors related to how the provider is situated with respect to inviting or disinviting interactions with clients. Attention to strategies at both the individual service provider and organizational levels is needed.

For example, an assessment of the organizational environment along the invitational continuum would use assessment prompts that are crafted specifically to focus on the organizational environment, broadly defined (Table 1) as an organizational environment that moves toward intentionally inviting care when practices are in place that affirm 2SLGBTQ+ people through the use of inclusive language on brochures and intake forms; ensure positive images of 2SLGBTQ+ people on the organization’s website and written materials; hire openly self-identified 2SLGBTQ+ employees across all levels of the organization; and engage in 2SLGBTQ+ health equity advocacy, among other practices. In the absence of all or some of these practices, services users may experience an organization as intentionally disinviting or unintentionally disinviting, prompting their perception of discriminatory care and harmful care interactions and environments.

#### 2.3.2. Implementing the 2SLGBTQ+ Home Care Access and Equity Framework

In this section, we animate the A&E Framework by drawing on three key findings from the project: (1) 2SLGBTQ+ invisibility in home care organizations; (2) lack of 2SLGBTQ+ training and education among home care service providers; and (3) avoidance of home care services by trans people. In doing so, we note the following points. First, we offer the key findings as examples of the ways in which the assessment prompts may be used to assess organizational practices related to community engagement, leadership, environment, policies and processes, education and training, and programs and services, as key indicators of access to care. Second, we note that the determination by an organization of their location on the invitational continuum should be an outcome of thorough and ongoing engagement with each indicator of access to care and a process characterized by reflection, dialogue, and consultation at all levels of the organization and involving key stakeholders. That is, an organization’s engagement with the indicators and assessment prompts does not offer a quantitative measure of the invitational continuum. Rather, through reflection, dialogue, and consultation, organizations may locate themselves along the continuum, revisiting the indicators and prompts to assess their progress over time. The process of organizational assessment is further explored below.

#### 2.3.3. 2SLGBTQ+ Invisibility in Home Care Organizations

Our interviews with senior home care administrators explored home care access and equity for 2SLGBTQ+ people from an organizational perspective. Our analysis of the data suggests a spectrum of 2SLGBTQ+ (in)visibility in home care organizations. In the worst-case scenario, 2SLGBTQ+ communities are not on the radars of organizations in terms of leadership, client care, or employee-related indicators. 2SLGBTQ+ people are invisible in areas such as strategic planning priority populations, orientation and continuing education, client complaints, and engagement with communities. 2SLGBTQ+ employees themselves are invisible. In some organizations, queer and trans people were vaguely visible, where a 2SLGBTQ+-related focus was found in one or a few aspects of the organization’s programs and policies, or there was some indication of community engagement. In the middle-ground scenario, queer and trans people were vaguely visible, such that there was some focus in orientation and continuing education, some client complaints, some engagement with 2SLGBTQ+ communities, and queer and trans employees were mostly invisible. In the best-case scenario, active steps were being taken by the organization to address the invisibility of 2SLGBTQ+ people. In these organizations, queer and trans service users were recognized as diverse, whether or not they were identified as priority populations. Service providers managed to document sexual and gender identities in affirming ways despite the absence of these on intake and assessment forms. In the best-case scenario, we saw positive signs of 2SLGBTQ+ visibility, in areas such as strategic planning priority populations, some orientation and continuing education, some focused care discussions, some client complaints, some engagement with communities, and 2SLGBTQ+ employees themselves were more visible. While this best-case scenario reflects promising practices to build from, efforts were still minimal, one-off initiatives in the absence of evidence that 2SLGBTQ+ equity work is meaningfully woven into the fabric of the organization.

This finding from our interviews with senior home care administrators in regional home care access centres is echoed in data from service user participants, which indicate that despite a need for formal home care services, only 40% of 2SLGBTQ+ service users reported that they had ever heard of home care access centres. Similarly, it is reflected in the service provider participant finding that 35% of home care service providers indicated that, as far as they knew, they had never worked with a 2SLGBTTQ+ client. Certain provider groups were more or less likely than others to report that they had never worked with a queer or trans client. A greater number of personal support workers, and fewer social workers and occupational therapists, stated that they had never worked with these clients. For those who had worked with a least one queer or trans client (65%), we asked how they came to find out about their clients’ sexual and gender identities. Similar to what we heard from service users, 85% of service providers indicated that their clients had self-disclosed.

This key finding suggests that regional home care access centres could use the A&E Framework to assess the degree to which they meaningfully engage, recognize, and are inclusive of 2SLGBTQ+ communities in the day-to-day operations of home care service delivery using the following access and equity indicators and assessment prompts: community engagement, leadership, environment, and policies and processes (see Table 2). As one home care administrator noted:

“It takes more than just having a few committed individuals. You really need to formalize [that within] an organizational structure, so you need to have a policy framework ensuring that you’ve got components of recognition of diversity built in, not just a singular policy on anti-discrimination but making sure that it’s woven through all of the policies … and, it really needs to be informed by the communities. (home care administrator)”

#### 2.3.4. Lack of 2SLGBTQ+ Education and Training among Home Care Service Providers

Consistent with what we know about who provides the bulk of the home care services in Ontario, we had more nurses, care coordinators, and personal support workers participate in this study than therapists (e.g., physio, speech language). As indicated above, it is clear that many home care service providers do not know they are working with sexual and gender minority clients. Providers do not ask about sexual and gender identities, but clients do tell. Certainly this lack of knowledge about working with 2SLGBTQ+ people may be related to lack of comprehensive 2SLGBTQ+-affirmative education and training in home care and in health provider curricula. Are home care service providers ready to receive their clients’ disclosures? Do they know what to do with this information?

While employed in home care, almost 90% of services providers had never received 2SLGBTQ+-focused education. Very few providers received 2SLGBTQ+-focused content as part of their continuing education. Our survey of 379 service providers indicated that, while employed in home care, 33% had attended at least 1 workshop with *some* 2SLGBTQ+ content. Often, these workshops were not always required (60%) and varied in length from 15 min or less (40%) to 30–60 min (60%). Only 13% of providers attended a workshop that focused exclusively on 2SLGBTQ+ content while employed in home care; 50% indicated that the workshop lasted 1–2 h. The content covered in the workshops was generally basic (e.g., definition of terms), focused on stigma and LGB people and to a much lesser extent, stigma and Two-Spirit and trans people. More specific issues related to 2SLGBTQ+ aging, HIV/AIDS, and end of life (palliative care) were discussed by several home care service providers as important continuing education needs. Importantly, personal support workers, who provide the bulk of home care services, were the least likely to have access to 2SLGBTQ+ education and training. In short, the few continuing education and training opportunities available to home care service providers were dedicated to information-sharing at the expense of practice skills, such as incorporating clients’ sexual and gender identities into care plans.

Importantly, because most service providers do not have education or training in this area, we heard over and over again that service users are put in a position of, or bear the burden of responsibility in, educating their providers:

“I had to educate, and they actually appreciated the education because they didn’t really have much experience with transgender people. They didn’t understand what it meant, so I had to explain it. (trans service user)”

Of particular note, there is a need for 2SLGBTQ+ education and training that underscores 2SLGBTQ+ communities as heterogeneous given intersections between sexuality and gender identity and race, class, and ability, among other social identities and locations. This requires home care organizations to not only consider how 2SLGBTQ+ people are involved in the development of educational material, but which 2SLGBTQ+ people are involved in these endeavours. The excerpt below not only indicates the additional emotional and labour burden put on racialized queer and trans service users to educate service providers on sexual orientation and gender identity, but also race, racialization, and racism:

“I’m done at this point of teaching people what Black means to me… there’s lots of books and the Internet and I can show you some resources, but I’m not going to be the person who’s always rehashing these parts of myself for your education. (queer service user)”

More specifically, this excerpt raises critical questions about whether and how home care organizations commit to education and training through an intersectional lens [57] to consider interlocking oppressions [4]—such as homophobia and transphobia *and* racism and anti-Black racism—as they are expressed through health disparities and inequitable access to high quality health care for Black, Indigenous, and people of colour (BIPOC). Health disparities that exist for BIPOC including higher risk for early onset of illness, a heavier burden of poor health, early death, mental illness, and chronic illness have been documented in the Global North [58]. While socioeconomic and environmental disadvantage is often linked to health disparities [59], mistrust of healthcare organizations and health professionals by BIPOC communities because of systemic racism is a significant contributor to such disparities [60,61]. Mistrust, a result of BIPOC communities being subjected to colonialism and racism during their health care interactions [23,62], leads to a refusal or reluctance to use of health care services, including preventative care [60,61]. Home care education and training must underscore health disparities caused by mistrust and fear that may exist for queer and trans BIPOC at the intersection of homophobia, transphobia, and racism/anti-Black and anti-Indigenous racism as a required practice consideration for service providers. Education and training must extend beyond the minimal benchmark of a provider’s racial “sensitivity” and “cultural competency” to ensure that all service providers know how to engage racialized queer and trans service users in ways that meaningfully take into account homophobia and transphobia and race and racism, among other acts of oppression, in their experiences of health/illness, and during health care interactions. Beyond the deleterious impact of bearing the responsibility of being involved in service provider education, in the absence of deeply critical education and training through an intersectional lens, racialized queer and trans home care service users are at risk from providers who lack education and training and rely on stereotypes about them in their practice.

It is critical to address both the lack of provider knowledge and understanding of 2SLGBTQ+ lives and communities generally, and the relevance of sexual and gender minority experiences to health and health services access specifically. To this end, home care organizations can assess their commitment to, resourcing of, and implementation of 2SLGBTQ+ continuing education and training for all employees using the A&E Framework indicator related to education and training. Particular considerations include ensuring that all employees, volunteers, and students have access to education and training opportunities; determining whether the organization is providing mandatory and ongoing initiatives rather than voluntary and one-off initiatives; whether education and training opportunities are provided during paid hours; how 2SLGBTQ+ people contribute to education and training programs; and ensuring that partner/contracted agencies meet 2SLGBTQ+ education and training requirements or expectations (see Table 3).

#### 2.3.5. Trans People Are Less Likely to Use Formal Home Care

Our study has confirmed the compelling research evidence that lack of service provider knowledge and clinical skills for trans people has a significantly negative impact on equity and accessibility of services for trans people. Service user participants told us that they prefer to avoid services when possible rather than expose themselves to discrimination or ignorance. In an example related to knowledge about transition-related surgery, one participant confirmed:

“The trans piece would be a knowledge gap for me; supporting the medical side of hormone therapy and all that. (Nurse)”

Undoubtedly, the nurse’s lack of education and training is a reflection of the overall lack of preparedness of, and investment by, the home care sector to provide affirming care to trans service users. As one trans woman explained:

“My health card still had “M” on it. They came in thinking they were dealing with a male. So that was kind of awkward for me. (trans woman)”

We interpret this key finding as an urgent call to develop specialized home care programs and services for trans service users. The A&E Framework can be used to assess the existence of 2SLGBTQ+ programming generally, and it can also be used to focus specifically on specialized programs and services such as those that prioritize trans inclusivity. For example, the A&E Framework can be used to assess whether trans people/staff are involved in programming and agency processes (e.g., strategic planning) and determine how the organization is involved in research to meet the goal of developing relevant trans programs and services (see Table 4).

As illustrated above, the A&E Framework is intended to centre organizational change on the unique and diverse realities of 2SLGBTQ+ service users while at the same time clearly operationalizing EDI and GBA+ concepts and goals. We offer examples of how the A&E tool may be used by an organization to assess the degree to which their programs and services are accessible to, and experienced by 2SLGBTQ+ service users as equitable and of high quality—that is, the degree to which programs and services are experienced by 2SLGBTQ+ communities as intentionally inviting, unintentionally inviting, unintentionally disinviting or intentionally disinviting. Of primary importance, however, is the identification of a clear series of steps that will facilitate the uptake and sustainability of the A&E Framework and its ongoing evaluation, as well as the ongoing identification and assessment of organizational EDI goals. This requires concretizing processes and strategies through mechanisms for implementation, assessment, and evaluation, and identifying desired outcomes to support equity and improved service accessibility for all 2SLGBTQ+ people. To this end, we offer the following steps for consideration derived from the *Diversity and Inclusion Framework and Implementation Plan* [63]:

1. Secure predictable and sustainable money resources;
2. Ensure committed and informed leadership through ongoing training. Attention must be paid to hiring members of equity-seeking groups into leadership positions;
3. Establish an equity, diversity, and inclusion (EDI) committee that includes staff across all levels of the organization, including out and diverse 2SLGBTQ+ staff, 2SLGBTQ+ community members that represent diverse experiences, knowledge, and perspectives, and other 2SLGBTQ+-relevant stakeholders (e.g., partner organizations);
4. Train all staff, including the EDI committee in order to build EDI capacity and competency and understanding of the A&E Framework;
5. Create a communication pathway and feedback loop to ensure that staff is regularly informed of, and can provide input on, A&E implementation goals, processes, and strategies;
6. Create a timeline and processes for A&E Framework implementation components including (to be completed by the equity consultant and EDI committee):
  - Determine a time period for the 2SLGBTQ+ A&E plan (e.g., 5-year plan with review and renewal every five years);
  - Identify organizational EDI goals and measures related to 2SLGBTQ+ access and equity, with realistic consideration of money and people resources (e.g., 15% of Board members identify as S2LGBTQ+ by [date]);
  - Prioritize change areas and change strategies (e.g., training for all service providers) based on the 6 key indicators;
  - Implement change strategies;
  - Determine an assessment and reporting timeline for the change strategies (e.g., quarterly assessment with an annual 2SLGBTQ+ A&E report);
  - Determine a timeline for the evaluation of the A&E Framework;
  - Determine an evaluation framework (e.g., audit of change strategies, consultation with equity advisors and EDI committee, 2SLGBTQ+ community, and other 2SLGBTQ+-relevant stakeholders);
  - Determine performance measures for the A&E Framework;
  - Revise A&E Framework where needed;
7. At the end of the 2SLGBTQ+ A&E plan period, assess organization change through an organization-wide process and identify EDI goals and measures related to 2SLGBTQ+ access and equity and change strategies for the subsequent plan period.

## 3. Concluding Thoughts

The Home Care Access and Equity Framework contributes to scholarship on 2SLGBTQ+ access and equity, providing a framework and process for organizational EDI change strategies within home care settings specifically and in health and social care service organizations generally. Within the Canadian health policy context, the A&E Framework constitutes a response to critiques of EDI and GBA+ by offering a specific 2SLGBTQ+ focus and measures to support 2SLGBTGQ+ engagement at the decision-making table. In addition, in the absence of EDI/GBA+ mandates within specific health sectors (e.g., provincial), the A&E Framework constitutes an accessible tool for 2SLGBTQ+ staff and allies in health care organizations as well as community activists and people with lived experience in their efforts to enact change. Even still, the cautions identified in the critical literature on GBA+ are worthy of repeating here, particularly since any access and equity initiative, no matter the framework, is subject to entrenched austerity, governmentality, and other mechanisms of neoliberalism, such as privatization and top–down management approaches to decision-making which have become increasingly normalized in health care design and delivery over the past decade. This means that community-based advocates and their allies in the health and social care sector must remain vigilant in their efforts to ensure that 2SLGBTQ+ people are included in all EDI/GBA+ mandates and initiatives, where they exist and to ensure 2SLGBTQ+ people become a priority target of inclusion where there is yet no explicit mandate. The invitational continuum and six indicators of access to care and assessment prompts that constitute the A&E Framework are key to enacting a strategy for the development of an inclusive, comprehensive, and systematic approach to organizational change. By adopting the A&E Framework introduced in this paper, health and social care organizations are provided with a roadmap that can both complement and supplement the EDI/GBA+ model.

## Figures and Tables

**Table 1:** Organizational assessment of environment.

Indicator of Access to Care	Assessment Prompts
Environment	How does staff use language to convey recognition, acceptance, and affirmation of 2SLGBTQ+ people? Are 2SLGBTQ+ people able to see themselves represented in visual cues within the physical environment of the agency (rainbow/triangle symbols, 2SLGBTQ+ representation of available brochures/posters; 2SLGBTQ+-relevant brochures/posters, promotional materials for agency services and programs that are 2SLGBTQ+-inclusive) and within/amongst agency staff? Do program-specific intake forms include demographic options that convey recognition, acceptance, and affirmation of 2SLGBTQ+ people? (Gender-neutral options such as ‘domestic partner’ or ‘same-sex partner’ along with gender-inclusive options to choose male/female/trans/trans man/trans woman/genderqueer, and gender-neutral questions about relationships and sexual behaviour) What processes are in place to foster the creation of culturally safe space and organizational support of diverse 2SLGBTQ+ employees? How does the agency administratively support 2SLGBTQ+ employee networks/working groups? Does the agency advocate for health equity initiatives to address systemic disparities in access? For example, does the agency advocate for community-based research that will increase their knowledge about the issues in providing 2SLGBTQ+-inclusive services and programs to diversely situated and identified populations?

**Table 2:** Assessing 2SLGBTQ+ (in)visibility.

Indicators of Access to Care	Assessment Prompts
Community Engagement	Are 2SLGBTQ+ people engaged with the agency? If so, who is represented? Does the community engagement process include diverse 2SLGBTQ+ experiences, knowledge, and perspectives? How are 2SLGBTQ+ people involved in needs assessment processes, identifying program/service directions, and delivering and evaluating them?
Leadership	Are openly 2SLGBTQ+ people in positions of leadership including within senior management? Is shared or distributive leadership used across the organization to engage in systems advocacy, for example, in relation to obtaining the resources (e.g., training or research) required to address health disparities and access barriers?
Environment	Do program-specific intake forms include demographic options that convey recognition, acceptance, and affirmation of 2SLGBTQ+ people (options such as “domestic partner(s)” or “same-sex partner(s)” and options to indicate used pronouns)? How does the agency administratively support 2SLGBTQ+ employee networks/working groups?
Policies & Processes	Are policies that address equity for both 2SLGBTQ+ service users and employees consistent across all programs? Do hiring practices include assessing diversity and 2SLGBTQ+ competence of candidates? Do staff evaluation practices include assessing diversity and 2SLGBTQ+ competency?

**Table 3:** Assessing home care provider 2SLGBTQ+ knowledge.

Indicator of Access to Care	Assessment Prompts
Education and training	Do all staff receive education and training? Where does training occur inside/outside the agency? What training opportunities occur (orientation, in-services, continuing education, specialized training to build clinical and human resources capacity)? How are 2SLGBTQ+ people involved in education and the development of material (e.g., train the trainer?)? How are diverse 2SLGBTQ+ experiences, knowledges, and perspectives reflected in education and training? How is education framed in terms of meeting agency goals for quality (mandatory/elective) accreditation? How does the agency culture support the notion of a learning organization, allowing for critical questioning of its practices?

**Table 4:** Assessing high-quality and equitable service for trans people.

Indicator of Access to Care	Assessment Prompts
Programs & Services	Are trans service users and staff involved in programming, agency processes (e.g., strategic planning)? How does the agency represent the voice/visibility of trans service users in programs and services? How are all programs inclusive of LGBTTQI health (e.g., intake forms)? Are programs developed, delivered, evaluated with trans communities? What reporting processes for clients/staff are in place to respond to discrimination, quality of care/work environment? How is the agency involved in research and advocacy to meet the needs of trans service users across, e.g., race, age, and condition?
